# Non-traumatic arm, neck and shoulder complaints: prevalence, course and prognosis in a Dutch university population

**DOI:** 10.1186/1471-2474-14-8

**Published:** 2013-01-04

**Authors:** Vivian EJ Bruls, Caroline HG Bastiaenen, Rob A de Bie

**Affiliations:** 1 Department of Epidemiology, Centre for Evidence Based Physiotherapy and Caphri Research Institute, Maastricht University, Maastricht, The Netherlands

**Keywords:** CANS, Upper extremity musculoskeletal disorders, Prevalence, Prognostic factors, Course, Cohort study, Survey

## Abstract

**Background:**

Complaints of arm, neck and shoulder are a major health problem in Western societies and a huge economic burden due to sickness absence and health-care costs. In 2003 the 12-month prevalence’s in the Netherlands were estimated at 31.4% for neck pain, 30.3% for shoulder pain, and 17.5% for wrist and hand pain. Research data suggest that these complaints are increasingly common among university students. The aims of the present study are to provide insight into the prevalence of complaints of arm, neck or shoulder in a university population, to evaluate the clinical course of these complaints and to identify prognostic factors which influence this course.

**Methods:**

The present study is designed as a prospective cohort study, in which a cross-sectional survey is embedded. A self-administered cross-sectional survey will be conducted to gain insight into the prevalence of complaints of arm, neck or shoulder among university students and staff, and to identify persons who are eligible for follow up in the prognostic cohort study. Patients with a new complaint of pain and discomfort in neck and upper extremities between 18–65 years will be asked to participate in the prognostic cohort study. At baseline, after 6, 12, 26 and 52 weeks individual patient data will be collected by means of digitized self-administered questionnaires. The following putative prognostic determinants will be investigated: socio-demographic factors, work-related factors, complaint characteristics, physical activity and psychosocial factors.

The primary outcome is subjective recovery. Secondary outcomes are functional limitations of the arm, neck, shoulder and hand, and complaint severity during the previous week.

**Discussion:**

To our knowledge, this is the first prognostic study on the course of complaints of arm, neck or shoulder that is conducted within a university population. Moreover, there are hardly any studies that have estimated the prevalence of these complaints among university students. The results of this study can be used for patient education and management decisions, as well as for the development of interventions. Moreover, identification of high risk groups in the population is needed to generate hypotheses or explanations of health differences and for the design of prevention programs.

## Background

Complaints of the arm, neck and shoulder (CANS) are a major health problem in Western societies [1,2]. Reported symptoms may include pain, clumsiness, tingling, stiffness, loss of coordination or physical strength, skin discoloration and temperature differences located in the neck, shoulder, arm, elbow, wrist, hand and/or fingers [3]. Not only are these complaints a major cause of severe long-term pain and physical disability, they also create a huge economic burden due to sickness absence and health-care costs [1]. In 2003 the 12-month prevalence in the Netherlands was estimated at 31.4% for neck pain, 30.3% for shoulder pain, 11.2% for elbow pain, and 17.5% for wrist and hand pain [4].

A population that receives relatively little attention with regard to CANS is the university student population. Although there are only few studies that estimated the prevalence of upper extremity symptoms among graduate or college students [5,6], research data suggest that frequent episodes of upper extremity symptoms are also prevalent among students [6]. The association of upper extremity symptoms with prolonged computer work [7,8], and the increasing use of computers by young workers and university students are reasons for concern that this population may be at risk for disabling musculoskeletal disorders. As university students are more and more entering computer-intensive occupations following graduation, complaints of arm, neck or shoulder may have significant effects on students’ professional career plans and productivity.

The high prevalence rates and the consequences of these complaints on well-being, career and productivity highlight the importance to gain insight into the frequency of these complaints among specific risk groups such as university students, as well as into the course of these complaints and their prognostic factors. However, studies on the course of CANS and the prognostic determinants are scarce, especially in a working population and among students.

Until now, studies on course and prognosis of upper limb musculoskeletal complaints show a large variety in patient characteristics and settings. Moreover, most studies have focused on a single site in the upper limb (for example, shoulder pain) [9]. Although these studies may provide useful clinical information concerning prognostic factors for local pain, recent studies carry implications that prognostic factors may be different for pain at multiple sites than for local pain at a single site in the upper limb [9]. Picavet et al. report that the majority of those reporting pain, report pain at more than one site within an anatomical area [1]. In addition, Walker-Bone et al. observed that neck and upper limb pain commonly cluster, and frequently display symmetry and adjacent patterns of involvement [9]. Given these findings, it is questionable whether the results of prognostic studies which focus on local pain may be extrapolated to pain at multiple sites at the upper extremity. So far, few long-term prognostic studies have focused on pain or symptoms at multiple sites of the upper extremity and neck simultaneously [10-13]. Karels et al. [11] conducted a prognostic cohort study in physiotherapy practice and included patients with complaints at multiple sites of the upper limb and neck. They reported that psychosocial variables as well as a long duration of complaints at baseline were negatively associated with recovery at six months. Feleus et al. [12], in a study performed in general practice, observed that characteristics such as long duration of the complaints or recurrent complaints were most predictive for non-recovery of complaints of arm, neck and/or shoulder 6 months after initial consultation.

Occupational factors, such as job demands and decision authority, have not often been taken into account although these factors have been shown to be related to outcome in research of work-related risk factors for low back pain [14]. Knowledge is lacking too concerning prognostic variables like pain at multiple anatomical sites and occupational factors on the long term. However, studies in neck and upper extremity complaints that did include pain at multiple anatomical sites and occupational factors were mainly executed in primary care setting [10-13]. Studies performed in occupational settings are scarce [15-17], and to our knowledge, a study on course and prognosis of CANS has never been conducted in a population of university students. Furthermore, knowledge is lacking with regard to information on prevalence of these complaints in university employees and students.

Therefore, the primary aims of the present study are:

1. To provide insight into the prevalence of CANS among a population of university staff and students.

2. To evaluate the clinical course of non-traumatic arm, neck and/or shoulder complaints in university staff and students and to identify prognostic factors which influence the course of non-traumatic arm, neck and/or shoulder complaints in this population.

## Methods

### Design and setting

**Figure 1:**
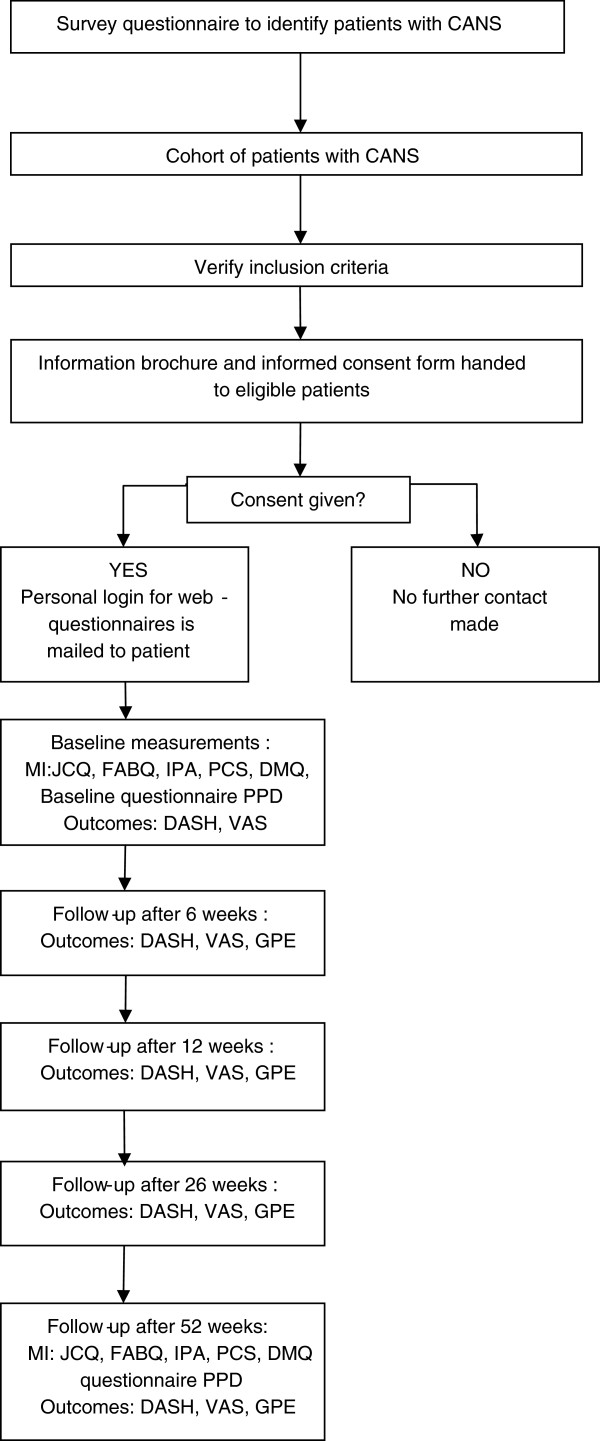
**Flow chart of the prospective cohort study.** CANS = Complaints of Arm, Neck or Shoulder; MI = Measurement instrument; JCQ = Job Content Questionnaire; FABQ = Fear Avoidance Beliefs Questionnaire; PCS = Pain Catastrophizing Scale; DMQ = Dutch Musculoskeletal Questionnaire; IPA = Impact on Participation and Autonomy questionnaire; PPD = putative prognostic determinants; DASH = Disability of Arm Shoulder and Hand Questionnaire; VAS = Visual analogue scale (complaint severity); GPE = Global Perceived Effect.

The present study is designed as a prospective cohort study (CANS Cohort Study), in which a cross-sectional survey is embedded. This study will be performed in the South region of the Netherlands. Recruitment of the study population will take place at two universities in this region: Maastricht University and Zuyd University of Applied Sciences. The base population (employees and students) of Maastricht University and Zuyd University of Applied Sciences consists of approximately 40,000 people (approximately 5,600 employees and 34,400 registered students). Study design and participant flow are shown in Figure 1.

With regard to the prospective cohort study, at baseline, and after 6, 12, 26 and 52 weeks, individual patient data will be collected by means of digitized self-administered questionnaires on patient level. The Medical Ethics Committee of the Maastricht University Medical Centre^+^, the Netherlands, has approved the study protocol, procedures and informed consent. Written informed consent will be obtained from all patients.

### Study population

Employees or students with a new complaint or new episode of complaints of pain and discomfort in neck and upper extremities (neck, shoulder, upper arm, elbow, forearm, wrist and hand) between 18–65 years are eligible for participation in the study. An episode of complaint is considered ‘new’ if patients have not visited their health care professional for the same complaint during the preceding 6 months. Exclusion criteria are: complaints caused by acute trauma or by any systemic disease, malignancy, prosthesis, amputation, congenital defect or a co-morbidity causing severe disability in daily activities, and not being able to fill in Dutch or English questionnaires. Patients who are pregnant will also be excluded from the study. Patients can be included in the study only once.

### Inclusion procedure

To identify persons with CANS for participation in the prospective cohort study, all employees and students of both universities receive a survey questionnaire. The content and procedure of this survey will be explained in the following paragraph.

The survey questionnaire is accompanied by an invitation letter, in which the respondent is informed concerning the purpose of the survey and about the possibility to participate in the prospective cohort study, if appropriate. Respondents who report to experience CANS in the survey questionnaire are requested to fill out an additional question in which they are asked whether they are interested in participation in the prospective cohort study. If they show interest, permission is asked in the survey questionnaire to be approached by the researchers. Subsequently, the researcher checks the inclusion criteria, which will be obtained from the survey questionnaire. If the respondent meets the inclusion criteria, a patient information leaflet about the study and an informed consent form will be sent by post, including a free post return envelope. A non-response card is also included.

Patients are asked to fill out the informed consent form within one week, if they decide to participate in the follow up study. Patients who are unwilling to participate are asked to return the non-response card.

In order to increase the response rate, patients who have not returned the informed consent form or the non-response card within two weeks will receive a reminder by e-mail. After returning the informed consent form, the patient obtains a personalized code by e-mail and is able to login to the web-questionnaires. Patients are asked to fill out the baseline questionnaires within 10 days.

### Cross-sectional survey

A self-administered cross-sectional survey will be conducted to gain insight into the prevalence rates of CANS among university staff and students, and to identify persons with CANS who are eligible for follow up in the prospective cohort study. All employees and students of both universities receive the survey questionnaire, accompanied by an invitation letter, by e-mail. In the invitation letter, employees and students will be asked to provide written informed consent, which covered the usage of the questionnaire data. To realize smooth processing of the large amount of questionnaires and to guarantee smooth enrolment of participants in the cohort study, questionnaires will be sent in separate batches, each containing approximately 2500 questionnaires. After 3 weeks a reminder e-mail will be sent and after 6 weeks a second reminder will be sent by e-mail.

The survey questionnaire consists of general questions and health questions about musculoskeletal conditions in: (1) neck; (2) shoulder; (3) upper arm(s); (4) elbow(s); (5) lower arm(s); (6) wrist(s); (7) hand(s)/ finger(s). The questions about each area start with the following question: “Did you experience pain/ symptoms in ‘anatomic area’ for at least one week during the past 6 months?” If a person replies positively, the person is asked to answer additional questions concerning the complaints at the relevant anatomic area: questions on the duration and severity of the pain/symptoms, consequences for health care utilization, questions on work status and sickness absence. Moreover, a list of the complaints that are classified as specific CANS, according to the CANS model [18] will be included together with the question “Has a doctor or health professional diagnosed one or more of the following complaints during the past 12 months?” (yes/no).

In the questionnaire which will be sent to the student population, specific questions on employment status are replaced by questions on study load and additional part-time jobs.

The items included in the survey questionnaire are partly derived from the Maastricht Upper Extremity Questionnaire (MUEQ), which has been found to be a valid questionnaire to assess the occurrence and nature of CANS and work-related physical and psychological risk factors [19]. Items of the MUEQ (items 69 to 95) which refer to the frequency, nature and clinical manifestations (i.e. tingling, numbness, weakness, swelling, stiffness, fatigue, continues pain and change in skin colour or temperature) of the complaint are included in the survey questionnaire. As the remaining items of the MUEQ refer to potential psychosocial risk factors and physical work related risk factors (based on the Job Content Questionnaire (JCQ) [20] and the Dutch Musculoskeletal Questionnaire (DMQ) [21]) and these questionnaires will be administered in the follow up measurements of the prospective cohort study, these items are excluded from the survey questionnaire. Furthermore, screen positives will be asked to answer the following three questions on help seeking behaviour. The first question is: ‘Have you already sought help for these complaints or symptoms?’, if the person replies positive, the second question is ‘by whom?’ (general practitioner and/or occupational physician, physiotherapist or someone else). If the person replies negative, the following question is: ‘Do you have the intention to seek help for your pain/symptoms in the near future?’ Lastly, the respondents who are screened positive are asked whether they are interested in participation in the prospective cohort study. If the person replies positive, three additional questions on the exclusion-criteria for the follow-up study, and permission to be approached by the investigator for further information on this study, will be addressed. The working population will be defined as respondents who report to have a paid job of at least 12 hours. The questionnaire will be available in Dutch and English.

### Procedure of data collection in the prospective cohort study

A computer-based patient record system (CPR), has been custom-built by Fastguide® [22]. The CPR enables participating patients to fill in web-questionnaires for the study by means of a personal account. When the next measurement moment for the study approaches, participants will be reminded by e-mail to login. In order to increase the response rate, participants who have not completed the baseline questionnaires within 10 days, will be reminded by telephone or e-mail by the principal investigator.

### Baseline and follow-up questionnaires

Based on information derived from preceding studies [10-12], the influence of the following putative prognostic determinants on the course of the complaints will be assessed: socio-demographic factors, complaint characteristics (specific/non-specific CANS) (assessed by means of the questionnaire on putative prognostic determinants (PPD)) psychosocial factors (fear avoidance beliefs, catastrophizing, psychosocial job characteristics, and perceived handicap), physical workload, co-morbidity, work related factors, physical activity during leisure time and treatment. After one year follow up the questionnaires will be addressed complete again. Work related follow up questionnaires do not concern the students in the study population. The complete overview of the questionnaires is shown in Table 1[20,21,23-33].

**Table 1:** Content and timing of the questionnaires and outcome measurements

**Outcome measures**	**Assessment**	**Measured in questionnaire**				
		**at baseline**	**at 6 weeks**	**at 12 weeks**	**at 26 weeks**	**at 52 weeks**
Subjective recovery	Global Perceived Effect (GPE) [14,15]		X	X	X	X
Complaint specific functioning	Disability of Arm Shoulder and Hand Questionnaire (DASH) [13]	X	X	X	X	X
Severity of complaints in previous week	11-point numerical rating scale [16]	X	X	X	X	X
**Putative prognostic determinants**						
Fear-avoidance beliefs about physical activity and work	Fear Avoidance Beliefs Questionnaire (FABQ) [17]	X				X
Catastrophizing	Pain Catastrophizing Scale (PCS) [18-20]	X				X
Psychosocial job characteristics	Job Content Questionnaire (JCQ) [21]	X				X
Physical workload	Dutch Musculoskeletal Questionnaire (DMQ) [22,23]	X				X
Perceived handicap	Impact on Participation and Autonomy questionnaire (IPA) [32]	X				X
Socio-demographic factors	Age, sex, body mass index (calculated from self-reported weight and height), ethnicity (open question), right-/left- handedness, smoking behavior (smoking every day, smoking now and then, not smoking but previously every day, not smoking but previously now and then, never smoked), marital status (unmarried/never been married, married/living together, widow, divorced), pregnancy, having children below 5 years of age in the household, educational level and work status, number of working hours per week (paid activities), working < 3 years in current job, profession (open question), study activities (are you a fulltime student?)	X				X
Complaint characteristics	Questions on complaint characteristics (Did you experience the same complaints of arm, neck or shoulder during the last six months for at least one week? How long do you have these complaints? Did the complaints occur sudden or did they develop gradually? What do you think is the cause of your complaints? Has a doctor or any health professional ever diagnosed one or more of the following complaints? (A list of the complaints that are classified as specific CANS, according to the CANS model, will be included) Did you use any medication to relieve your complaints during the past three months (over the counter or prescription)? Do you have any other musculoskeletal complaints? Are you currently under treatment of a health-care professional for these complaints?	X				X
Co-morbidity	Question about co-morbidity (Are you familiar with other diseases or disorders besides the CANS?)	X				X
Work-related factors	Questions about work and sick leave due to CANS (Were you absent in the past six months because of CANS ? If yes, how many days were you absent because of CANS? Did you adapt your activities at work or study due to CANS? Do your complaints return or worsen during work or study? Do the complaints diminish after several days of work or study?)	X				X
Physical activity during leisure time	Questions about type of physical activity and how often (Do you participate in sports during one hour or more each week? Do you accumulate 30 minutes or more of moderate-intensity physical activity on at least five days of the week (Norm of Healthy Activity) [33]? How frequent do you perform the following physical activities: housekeeping, gardening, do-it-yourself work, computer use, playing a musical instrument, taking care of small children (< 5 yrs) or disabled persons and handcrafts (seldom/ sometimes/ always)	X				X

### Outcome measures

After baseline assessment, follow-up assessments will be conducted 6, 12, 26 and 52 weeks.

The primary outcome measure, subjective recovery, will be measured with the Global Perceived Effect (GPE) scale [24,25]. Patients are considered to be recovered when they report that they are much improved or fully recovered. Since there is no universally accepted definition of recovery from CANS and the GPE scale may not offer an accurate measure of change as transition time stretches into months [34], we will supplement GPE with two secondary outcomes: functional limitations and complaint severity during the previous week. First, the functional limitations of the arm, neck, shoulder and hand will be measured using the Disability of Arm Shoulder and Hand Questionnaire [23]. Using this self-report system, patients attribute scores of 1 to 5 on 30 items relating to functional activities and symptoms. Response scores will be summed and transferred to a score ranging from 0 (no disability) to 100 (completely disabled). Secondly, the severity of complaints in the previous week will be measured on a numerical rating scale from 0 (no pain/complaints) to 10 (intolerable pain/complaints) [26]. Patients are considered to be recovered when they report that they are much improved or fully recovered.

### Statistical analysis

The course of the complaints will be described by means of descriptive statistics. Determinants that might influence the clinical course will be established by means of multivariate logistic regression analysis and analysis for repeated measurements over time (GEE; Generalized Estimating Equations). In univariate analyses the putative prognostic variables for the persistence of complaints will be selected. These analyses will be done separately for the working and non-working subpopulations.

Outcome measures will be analyzed separately in different multiple regression analyses. Pearson or Spearman correlation coefficients will be used to calculate correlations between potential determinants. When a high correlation between two determinants is found, only the most predictive determinant in the univariate analyses will be included in the multiple regression models.

Prevalence rates of CANS over the past twelve months that lasted for at least one week will be computed including 95% confidence intervals (CI).

### Power

A recruited sample of 500 patients will be sufficient to detect prognostic indicators with at least 80% power, assuming a maximum of 20% loss to follow-up and 5% two-tailed significance level. The sample size is likely to be adequate for exploring the independent association of at least 15 prognostic indicators for better outcome. The base population (employees and students) of Maastricht University and Zuyd University of Applied Sciences consists of approximately 40,000 people. We need an enrolment of 0,8% of the total population for the prospective cohort study. Regarding the already mentioned prevalence rates of the complaints in the introduction it seems a realistic goal.

## Discussion

Little information is available on the course and prognostic factors for recovery in patients with CANS, as well as on the prevalence of CANS in a population of university staff and students. This study is designed to provide insight in the prevalence of CANS, its course and the long term prognosis in a university population.

An important strength of this study is the ability to present the natural (untreated) course of CANS, because not all employees and students that will be followed up in the prognostic cohort study, are under treatment for their complaints. Another strength is that the use of three domains of recovery (subjective recovery, functional limitations and complaint severity) allows to describe a broader perspective of relevant health outcomes for patients with CANS.

The study also has some limitations. Sample selection might have implications for the internal validity of the study, especially if there is selection bias at the moment of the survey. Because the goal of the present study is to invite all employees and students to complete the survey questionnaire (including those who are off work or study due to disabling musculoskeletal complaints), selective non-response is not likely to occur. A possible threat for the internal validity of the study concerns the issue of selective withdrawal. Selective withdrawal might occur in case of the self-administered survey questionnaire or the questionnaires of the cohort study. This might be due to the possibility that respondents or participants with relatively severe CANS are not able or willing to complete digitized questionnaires because of their pain or symptoms. Furthermore, employees or students with severe CANS may have already left the labour force or discontinued their study. This may lead to an underestimation of severe CANS in the university population. Consequently, a higher prevalence of people with severe CANS would possibly be observed in the general population.

On the contrary, it is also possible that people with complaints are more willing to participate than those without problems. This may lead to a slight overestimation.

Moreover, in this cohort study the complaints will be divided into specific and non-specific diagnosis based on the CANS-model [18]. According to this model, 23 conditions were classified as specific disorders. All other conditions were labeled as non-specific CANS in this model. The classification into specific and non-specific in this study is based on self reports. The assessment of disease information which is based on self reports is limited owing to undiagnosed diseases and false diagnosis, because the patient forgets on it or misunderstands the diagnosis. Because patients in this study do not undergo a standardized physical examination by a single observer, there may be some misclassification with regard to classification into specific and non-specific. On the other hand, for the assessment of diseases that are characterised by pain and functional limitation, it is often agreed that the individual subject is the single best source of information [1].

The results of the prospective Cohort Study (CANS cohort study) can be used for patient education and management decisions, as well as for the development of interventions for these complaints. Moreover, identification of high risk groups in the population is needed to generate hypotheses or explanations of health differences and for the design of prevention programs [4].

To our knowledge, this is the first prospective study on the course of CANS, and the factors that influence this course, that is conducted in a university population. Furthermore, there are hardly any studies that have estimated the prevalence of CANS among university students.

The results of this study will be presented as soon as they are available.

## Competing interests

The authors declared that they have no competing interest.

## Authors’ contributions

VEJB conceived of the project, led the design and co-ordinates the study, and wrote the draft of the manuscript. RADB and CHGB participated in the study design and commented on drafts of this paper. CHGB provided advice for the method section. All authors have reviewed and approved the final manuscript.

## Pre-publication history

The pre-publication history for this paper can be accessed here:

http://www.biomedcentral.com/1471-2474/14/8/prepub
